# Examining spatiotemporal dynamics of CO_2_ emission at multiscale based on nighttime light data

**DOI:** 10.1016/j.heliyon.2025.e41806

**Published:** 2025-01-08

**Authors:** Binbin Zhang, Zongzheng Liang, Wenru Guo, Zhanyou Cui, Deguang Li

**Affiliations:** a College of Sciences, Shihezi University, Shihezi, 832003, China; b Academy of Regional and Global Governance, Beijing Foreign Studies University, Beijing 100089, China; c School of Information Technology, Luoyang Normal University, Luoyang 471934, China; d Faculty of Engineering, Huanghe S&T University, Zhengzhou 450000, China

**Keywords:** CO_2_ emission, Spatiotemporal dynamics, Nighttime light data, DMSP-OLS, Linear regression model

## Abstract

Carbon emissions have increasingly been the focus of all governments as countries throughout the world choose carbon neutrality as a national development strategy. The analysis of the spatiotemporal dynamics of CO_2_ emission has emerged as a significant research topic considering the dual-carbon goal. In this research, we explore the spatiotemporal changes of CO_2_ emission at different scales based on nighttime light data. The Chinese Academy of Science's Earth Luminous Dataset, CO_2_ emission data from Carbon Emission Accounts and Datasets, and basic national geographical data are used for analysis. A linear regression model between nighttime light data and CO_2_ emission is constructed. Thereafter, the global Moran's I index of exploratory spatial data analysis is used to verify the spatial parameters of all provinces. The trend value method is utilized to analyze the changing trend of CO_2_ emission at multiscale levels, covering the Chinese mainland, three major economic regions, and six largest agglomerations from 2012 to 2019. Experimental results show a significant positive correlation between the CO_2_ emission and nighttime light data from 2012 to 2019. The nighttime light data could be used to effectively estimate the total CO_2_ emission at the provincial and municipal levels in China. The growth rate of CO_2_ emissions in China is stable and has decreased in 2015. Furthermore, the spatiotemporal dynamics of CO_2_ emission in different agglomerations vary. Our work provides a scientific basis for the different provinces and cities to develop feasible emission reduction policies.

## 1 Introduction

The global climate has rapidly changed in recent years, and global warming has become increasingly serious. According to the report of “Global climate change for one to ten years” produced by the UK Met, 1 year with the period of 2021–2025 is likely to be the hottest year on record. CO_2_ emissions are regarded as the main factor that affect environmental changes, as fossil fuels used by human release a large amount of CO_2_, and the asymmetric development of high CO_2_ emissions and low absorption has become the main influencing factor of climate warming [1,2]. In fast-developing countries, the consumption of fossil energy and the growth of carbon emissions are inevitable [3]. Large amounts of CO_2_ emissions have restrained the sustainable development process of China due to the advancement of the country's industrialization and urbanization since the reform and opening in 1978 [[4], [5], [6]]. According to statistics from the United Nations, China has become the largest CO_2_ emission country in 2010 [7]. Despite this situation, the country pays great attention to the issue of carbon emissions, actively responds to climate change, promises to reduce carbon emissions per unit of GDP by 60 % by 2030 compared with 2015, and strives to reach the peak of CO_2_ emissions by 2030 and achieve carbon neutralization by 2060 [8]. China is under great pressure to save energy and reduce carbon emissions. Moreover, the energy consumption structure, economic development model, and urban–rural transformation process in different regions of China greatly vary. The spatiotemporal characteristics and changes of CO_2_ emissions in different regions of the country must be scientifically and accurately measured and analyzed to formulate reasonable energy saving and emission reduction plans.

Numerous scholars have conducted research on the changes in carbon emissions [[9], [10], [11]]. Majority of the studies use statistical data of administrative units as data sources. Research on CO_2_ emissions in China encompasses various perspectives, including the total amount of national energy consumption changes, the CO_2_ emissions at the national scale, the spatial pattern of CO_2_ emissions, the change trend of per capita carbon emissions, the spatial pattern of energy consumption, and the disparities in carbon emissions across different industries [[12], [13], [14], [15], [16], [17], [18], [19]]. Moreover, studies examine the main factors affecting CO_2_ emissions by combining energy consumption data, population data, GDP, and other data [[20], [21], [22]]. In addition, different mathematical models are used to analyze the leading factors affecting carbon emissions in China, reveal the contribution rates of the influencing factors of carbon emissions in different regions, and conduct research on carbon emission reduction strategies aiming at the leading factors [[23], [24], [25], [26]]. Clark-Sather et al. [27] estimated the CO_2_ emissions in China at the provincial scale during 1997–2007. Cheng et al. [28] analyzed the spatiotemporal dynamics of domestic CO_2_ emissions during 1997–2012 b y using logarithmic and Kaya equations. Gingrich et al. [29] calculated the CO_2_ emissions from fossil fuels in foreign cities from 1832 to 2000. Dong [30] studied the drivers of the decoupling indicator between economic growth and energy-related CO_2_ emission in China from the perspectives of decomposition and spatiotemporal heterogeneity. However, most studies are mainly based on the official statistical data of different units. Consequently, data inconsistencies and clutter problems arise due to differences in statistical errors and calculation methods at various levels. Therefore, a new accurate method is needed to estimate China's carbon emissions by using remote sensing technology.

Remote sensing technology has gradually become an important way to detect the spatiotemporal changes in geography with rapid development. Remote sensing satellites could comprehensively monitor the temporal and spatial dynamics of human society. Take the operational line-scan sensor (OLS) carried by the defense meteorological satellite program (DMSP) of the United States as an example, the nighttime light data obtained could effectively reflect the effects of human activities [[31], [32], [33], [34], [35], [36]]. Researchers across the world have used DMSP/OLS nighttime light data for urbanization monitoring [37], economic growth assessment [[38], [39], [40]], and power and other energy consumption estimation [41,42]. Numerous scholars have explored the correlation between nighttime light data and CO_2_ emissions and established different regression models to estimate CO_2_ emissions. Elvidge et al. [43] conducted a correlation analysis on nighttime light and power consumption data, GDP data, and CO_2_ emission data of 21 countries at the global level and found that nighttime light data were correlated with these data. Doll et al. [44] conducted a correlation analysis on DMSP/OLS nighttime light and CO_2_ emission data in 46 countries at the global level. The results showed that nighttime light data had a significant correlation with CO_2_ emissions, indicating that fitting spatial distribution with CO_2_ emissions by nighttime light data was effective. Meng et al. [45] proposed a method to draw spatiotemporal characteristic maps of CO_2_ emissions by using DMSP/OLS and CO_2_ emission data at the national and urban levels. Shi et al. [46] established a multi-scale panel model for CO_2_ emission estimation in China, inverted CO_2_ emissions to county scale, and conducted spatial econometric analysis. The results proved that a significant positive linear correlation exists between nighttime light data and CO_2_ emissions. Lu et al. [47] conducted a correlation analysis and spatiotemporal characteristics between DMSP/OLS and human activity intensity at the country level. Su et al. [48] proposed a model to estimate CO_2_ emissions at the provincial scale by using DMSP/OLS data and land remote sensing data and feasible emission reduction strategies. Chen et al. [49] presented a coupled decomposition analysis of CO_2_ emissions due to energy consumption in China and analyzed the driving factors of CO_2_ emissions in eight sub-periods over 1995 to 2011. Liu et al. [50] proposed an improved disaggregating model based on vegetation adjusted nighttime light data to estimate the spatiotemporal variations of city-level energy-related CO_2_ emissions. Shi et al. [51] combined DMSP-OLS nighttime light images, statistical energy consumption data, and urban area data to assess the spatiotemporal variations of urban CO_2_ emissions in China from national scale down to regional and urban agglomeration scales between 1997 and 2012. Zhao et al. [52] examined the spatiotemporal dynamics of urban residential CO_2_ emissions and their driving forces in China by using the integrated DMSP-OLS and VIIRS nighttime light datasets to estimate the CO_2_ emissions of urban residential from 2000 to 2015. Zhou et al. [53] examined the determinants and the spatial nexus of city-level CO_2_ emissions in China from 1992 to 2013. Lv et al. [54] conducted a multiscale analysis on the spatiotemporal dynamics of energy consumption CO_2_ emissions in China by utilizing the integrated DMSP-OLS and NPP-VIIRS nighttime light datasets from 1992 to 2016.

Most previous studies use traditional statistical methods, and the research dates are concentrated in earlier periods. Meanwhile, research on simulating CO_2_ emissions using nighttime light data is still in the preliminary stage. Analyzing the spatiotemporal dynamics of CO_2_ emission, accurately quantifying the total CO_2_ emissions in different regions of the country, and formulating reasonable emission-reduction and energy-saving policies for the various regions of the country have become worthy research topics. Accordingly, we use the data of cities at all levels to calculate the CO_2_ emissions according to the IPCC model for calculating CO_2_ emissions from energy consumption based on the flint dataset provided by the Chinese Academy of Sciences Remote Sensing Satellite Earth Station and the carbon emission data released by the China Carbon Accounting Database. Thereafter, we build a linear relationship model between lighting data and CO_2_ emissions.

The global Moran's I index of the exploratory spatial data analysis is used to test the spatial attributes of all provinces and cities. The trend value method is used to analyze the change trend of emission. Finally, the spatiotemporal characteristics of CO_2_ emissions in China during the 2012–2019 years are analyzed at the multiscale levels. The temporal and spatial characteristics of the CO_2_ emissions from 2012 to 2019 in China are analyzed to provide an effective support for formulating differentiated emission reduction policies for different provinces, cities, and regions.

## 2 Study area and data

### 2.1 Study area

**Fig. 1:**
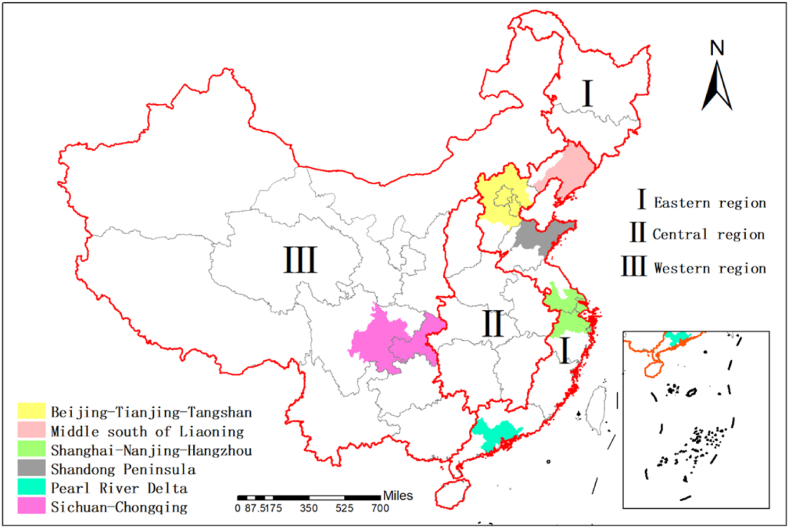
Location map of the study areas.

This work analyzes the spatiotemporal characteristics of the CO_2_ emissions at the national, regional, and urban agglomeration levels. From the national scale, the CO_2_ emission in China is mainly affected by GDP, population, urbanization, and other factors. In recent years, China's GDP has shown positive growth. In 2020, China's GDP reached 101.598 trillion yuan, and its population is 1.4 billion. The acceleration in economic and population growth has contributed to an increase in CO_2_ emissions. Consequently, the spatiotemporal dynamics of the CO_2_ emissions at the national level must be analyzed. The development speeds vary at the regional scale due to the large disparities in different regions of China. China is divided into three regions, namely, the eastern, central, and western regions, based on the characteristic of the physical geography and economic development (Fig. 1). Thereafter, the spatiotemporal variation of the CO_2_ emissions of the three regions are analyzed. In the past few decades, China promoted urbanization at an unprecedented speed. Six urban agglomerations gradually formed, concentrating a significant portion of China's population and economic activities. The amount of CO_2_ emission in these urban agglomerations is the main factor that constitutes the national CO_2_ emission. On this basis, this study selects the Pearl River Delta, Shanghai–Nanjing and Hangzhou, middle south of Liaoning, Sichuan–Chongqing region, Beijing–Tianjin–Tangshan, and Shandong Peninsula as six urban agglomerations to conduct research and analysis on the spatiotemporal changes of CO_2_ emissions [55]. The location map of the research area is shown in Fig. 1.

### 2.2 Data source

Two types of nighttime light data, namely, DMSP/OLS nighttime light image and Visible infrared Imaging Radiometer NPOESS Preparatory Project (NPP/VIIRS) nighttime light dataset, are commonly used by researchers. The DMSP/OLS data were collected with the OLS sensors of the U.S. military meteorological satellite program. Meanwhile, the dataset only continued until 2014, and the spatial resolution was considerably low. The NPP/VIIRS dataset is collected by the VIIRS sensor of the US's Suomi National Polar-Orbiting Partnership, which is a new generation of nighttime light dataset to overcome the shortcomings of the DMSP/OLS sensors. This work uses the Flint dataset provided by the China Remote Sensing Satellite Ground Station of the Chinese Academy of Sciences, which is an optimized dataset based on the U.S. Suomi-NPP satellite data. The Flint dataset has long data time series, smaller volume, higher light image quality, and other advantages. In this work, we use the beta1 version of the Flint dataset, which has a resolution of 1500 m, specifically 0.0125°/pixel (28,800 × 11,200 pixels).

The CO_2_ emissions in this work are from the China Carbon Accounting Database from 2012 to 2019 [56,57]. The statistical data on energy consumption in various provinces and prefecture-level cities were collected as expanded data. Meanwhile, the statistical data on coal, gasoline, kerosene, diesel, fuel oil, natural gas, coke, crude oil, and electricity in some provinces and prefecture-level cities were obtained. The CO_2_ emissions of the prefecture-level cities were calculated based on the energy calculation CO_2_ emissions formula in the 2006 greenhouse gas emission inventory provided by the Intergovernmental Panel on Climate Change (IPCC) (Equation (1)) [58,59]. This method for estimating CO_2_ emissions is based on literature[55,60,61]. Although this method provides different coefficients for converting different energy sources into CO_2_ emissions, some controllable errors in the calculation occurred due to the different types of energy sources and the interactions between them.

(1) $${\text{CO}}_{2}=\frac{44}{12}\times \sum_{i=1}^{9}K_{i}E_{i}\text{,}$$

where $K_{i}$ is the CO_2_ emission coefficient of energy i, $E_{i}$ is the consumption of energy i calculated based on the standard coal, and coefficient $\frac{44}{12}$ is the carbon proportional constant. Table 1 shows the CO_2_ emission coefficient of each energy converted into standard coal.

**Table 1:** Carbon emission factor for the different types of fuels.

Energy type	Coal	Gasoline	Kerosene	Diesel fuel	Fuel oil	Natural gas	Coke	Crude	Electricity
Standard coal conversion factor	0.7143	1.4714	1.4714	1.4517	1.4286	1.33	0.9714	1.4286	0.345
Carbon emission factor	0.7559	0.5538	0.5714	0.5921	0.6185	0.4483	0.855	0.5857	0.272

Note: unit of standard coal conversion coefficient (t standard coal/t); the unit of electric power conversion coefficient is kg/kWh; and the unit of CO_2_ emission coefficient is ({10^4^t carbon/10^4^t standard coal).

The geographic information data used in this work mainly include national administrative division, provincial vector administrative boundaries, municipal vector administrative boundaries, main islands, and adjacent sea areas. This information comes from 1:1,000,000 basic geographic data released by the national catalogue service for geographic information. Table 2 shows some key information of the dataset used in this work.

**Table 2:** Description of dataset.

Data	Data description	Year	Source
Nighttime light	Flint beta1 (28,800 × 11,200 pixels)	2012–2019	http://satsee.radi.ac.cn/cfimage/nightlight/
CO_2_ emission data	Carbon Emission Accounts and Datasets	2012–2019	https://www.ceads.net/data/province/
Geographic coordinates	National basic geographic data (1:1,000,000)	–	https://www.webmap.cn/main.do?method=index

## 3 Research methods

### 3.1 Model and correlation analysis

Before fitting the nighttime light data and CO_2_ emission data, the correlation of the two variables should be evaluated first. Correlation usually has three kinds of relations, positive correlation, negative correlation and uncorrelation. A positive correlation refers to the mutual correspondence between the high and the low data values of two variables. Negative correlation refers to the high value of one variable that corresponds to the low value of the other. Uncorrelation refers to the absence of relationship between two variables. The change of one variable has no effect on the other. The commonly used coefficient for correlation analysis among variables is Pearson correlation coefficient. The calculation is shown in Equation (2).

(2) $$r_{X,Y}=\frac{Cov\left(X,Y\right)}{\sqrt{Var\left[X\right]Var\left[Y\right]}}\text{,}$$

where Cov(X,Y) is the covariance of X and Y, Var[X] is the variance of X, and Var[Y] is the variance of Y. When the value of $r_{X,Y}$ approaches 1 or −1, a strong correlation exists between X and Y. In the process of building the relation between nighttime light data and CO_2_ emission, the total value of nighttime light of each province should be counted first. Thereafter, the fitting analysis is carried out with the CO_2_ emission statistics of the province. The fitting linear model is shown in Equation (3).

(3) $$E_{i}=aS_{i}+b\text{,}$$

where $E_{i}$ is the estimated CO_2_ emission of pixel i of the research map, ${\;S}_{i\;}$ is the light value of pixel i, and a and b are the regression coefficients. This work conducts a correlation analysis based on the lighting and emission data of 34 provinces and cities in China during 2012–2019. The correlation analysis results and the comparison between the estimated value and the real value of CO_2_ emission based on the model are shown in Fig. 4 and Table 5.

### 3.2 Evaluation model for the spatiotemporal dynamics

To better evaluate the spatiotemporal dynamics of the CO_2_ emissions, this work uses Moran's I index to test the overall spatial attributes of provinces and regions and the trend value method to analyze the change of CO_2_ emissions. Exploratory spatial data analysis (ESDA) refers to the use of statistics and geography and other theories to identify the spatial data attributes by using spatial relationships as a measure and study the variation of the global spatial relationship and local autocorrelation. In this work, global Moran's I index is used to analyze the spatial correlation degree and overall spatial attribute of the CO_2_ emissions in China. The index could effectively reflect the overall regional aggregation when the value of Moran's I index tends to 1 or −1. This index indicates strong similarity between adjacent spatial units. The specific calculation is shown in Equation (4).

$$I=\frac{n\sum_{i=1}^{n}\sum_{j=1}^{n}W_{ij}\left(x_{i}-\bar{x}\right)\left(x_{j}-\bar{x}\right)}{\left(\sum_{i=1}^{n}\sum_{j=1}^{n}W_{ij}\right)\sum_{i=1}^{n}{\left(x_{j}-\bar{x}\right)}^{2}}\text{,}$$

$$\bar{x}=\frac{1}{n}\sum_{i=1}^{n}x_{i}\text{,}$$

(4) $$\left\{ \begin{array}{c} W_{ij}=\frac{1}{d_{ij}}\;i\neq j\\ W_{ij}=0\;i=j \end{array}\right.\text{,}$$

where $x_{i}$ represents the CO_2_ emissions of i province, $x_{j}$ represents the CO_2_ emissions of j province, n represents the number of provinces and cities, $W_{ij}$ represents the spatial weight matrix, and $d_{ij}$ represents the surface distance calculated by latitude and longitude between provinces and cities.

Slope method refers to the use of univariate phase regression model to calculate the change slope of time span and analyzes the linear tendency value of CO_2_ emission in each city. The calculation is shown as Equation (5).

(5) $$\text{SLOPE}=\frac{n\sum_{i=1}^{n}x_{i}C_{i}-\sum_{i=1}^{n}x_{i}\sum_{i=1}^{n}C_{i}}{n\sum_{i=1}^{n}{x_{i}}^{2}-{\left(\sum_{i=1}^{n}x_{i}\right)}^{2}}\text{,}$$

where n is the number of years, $x_{i}$ is the i year, and $C_{i}$ is CO_2_ emissions of the i year. The corresponding grade classification is shown in Table 3. The grade classification in Table 3 is based on the actual statistical values of CO_2_ emissions from 2012 to 2019, which is divided into five levels: low, relatively low, medium, relatively high and high. The classification of CO_2_ emission can be more comprehensively and carefully studied by using the tendency method.

**Table 3:** Classification criteria of the CO_2_ emission SLOPE.

Growth type	Low	Relatively low	Medium	Relatively high	High
SLOPE value	$\langle \bar{C}-0.5\;s$	$\bar{C}-0.5\;s∼\bar{C}+0.5s$	$\bar{C}+0.5\;s∼\bar{C}+1.5\;s$	$\bar{C}+1.5\;s∼\bar{C}+2.5\;s$	$>\bar{C}+2.5\;s$

Note: $\bar{C}$ is the average CO_2_ emission, and s is the standard deviation.

## 4 Results and discussion

### 4.1 Correlation analysis and 3D spatiotemporal map

#### 4.1.1 Correlation analysis

**Fig. 2:**
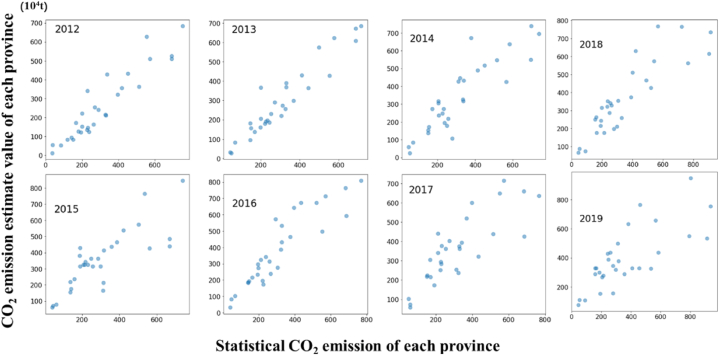
Statistical and estimated CO_2_ emissions by nighttime light data.

This study uses provincial CO_2_ emission and nighttime light data from 2012 to 2019 as the modeling data and adopts the ordinary least squares (OLS) method for modeling. Table 4 presents the OLS regression result of the established model. The goodness of fit of the model is 0.75, and the *P* value of the independent variable nighttime light data is less than 0.01, indicating that the data is significant at the 1 % level. The attribute values of the correlation analysis between the predicted CO_2_ emission data and the actual data are presented in Table 5. The Akaike Information Criterion (AIC) and Bayesian Information Criterion (BIC) presented in Table 4, Table 5 serve as valuable tools for model selection in OLS regression analysis. A lower AIC value indicates a superior model with stronger explanatory power, while a lower BIC value signifies a more robust fit between the model and the observed data. Fig. 2 shows a good linear correlation between the two variables, proving that the practical linear fitting model in this work is reasonable and effective.

**Table 4:** OLS regression result of our fitting model.

R-squared:	0.756	Omnibus:	2.219
Adj. R-squared:	0.755	Prob(Omnibus):	0.330
F-statistic:	459.8	Skew:	0.242
Prob (F-statistic):	3.00e−47	Kurtosis:	3.225
Log-likelihood:	−894.97	Durbin–Watson:	1.504
AIC:	1794.	Jarque–Bera (JB):	1.777
BIC:	1800.	Prob(JB):	0.411

	Coef	Std err	t	P>|t |	[0.025	0.975]
Intercept	66.7646	15.138	4.410	0.000	36.851	96.679
Light	0.8945	0.042	21.443	0.000	0.812	0.997

**Table 5:** Attribute values of the correlation analysis between the predicted CO_2_ emission data and the real data.

Import factor	2012	2013	2014	2015	2016	2017	2018	2019
$R^{2}$	0.874	0.905	0.790	0.666	0.831	0.694	0.778	0.58
AIC	338.1	331.0	359.0	367.5	358.1	361.7	361.3	381.9
BIC	340.9	333.8	361.8	370.3	360.9	364.5	364.1	384.7
Prob(JB)	0.0409	0.0306	0.149	0.856	0.612	0.862	0.339	0.409

#### 4.1.2 Comparison between the estimated and the statistical values

**Fig. 3:**
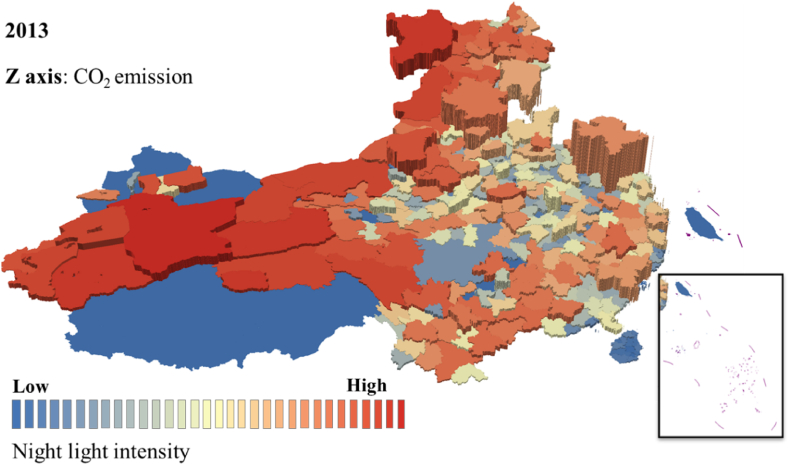
3D map of the nighttime light and CO_2_ emission in 2013.

**Fig. 4:**
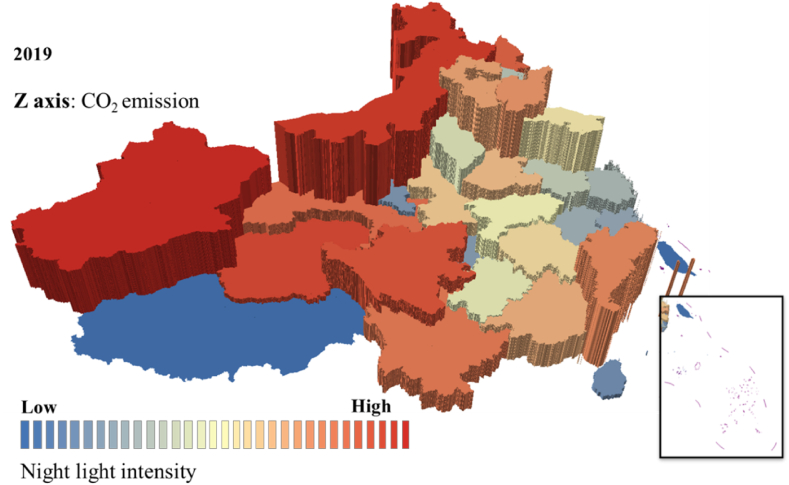
3D map of the nighttime light and CO_2_ emission in 2019.

The estimated value was compared with the real statistical value of the CO_2_ emission to verify the accuracy and reliability of CO_2_ emission estimated value obtained by linear fitting. The comparison result is shown in Fig. 2. Some attribute values of the correlation analysis are shown in Table 5. The root means square error of the CO_2_ emission estimated value and statistical real value is 9.4379 million tons, and the relative error is 7.56 %. Accordingly, the estimated CO_2_ emission obtained by this model has high precision and can be used to explore the dynamic change of CO_2_ emission. In addition, we draw the 3D spatiotemporal map of the two to further intuitively display the intuitive relationship between nighttime light and CO_2_ emissions, as shown in Fig. 3, Fig. 4.

#### 4.1.3 3D spatiotemporal map of the CO_2_ emission and nighttime light

Fig. 3, Fig. 4 shows the 3D spatiotemporal map of the nighttime light and CO_2_ emission, in which color is used to represent the data value of nighttime light, with the red area indicating higher nighttime time light value, and the blue portion representing the opposite. The height value of the z-axis represents CO_2_ emission. The higher z-axis means the larger CO_2_ emission. According to the nighttime light data and CO_2_ emission data from 2012 to 2019, the figures are made by accumulative statistics of the prefecture-level cities. The correlation between nighttime light and CO_2_ emission is high, and CO_2_ emission is relatively high in areas with high nighttime light intensity. However, the correlation in Jiangsu, Zhejiang, and Shanghai regions is weak, as coastal cities developed, which brings high CO_2_ emission with economic development while the nighttime light of these regions is stable, and the CO_2_ emission of these regions is decreased during these years.

### 4.2 Analysis of the spatiotemporal dynamics of the CO_2_ emissions

#### 4.2.1 CO_2_ emission spatiotemporal dynamics at the national scale

**Fig. 5:**
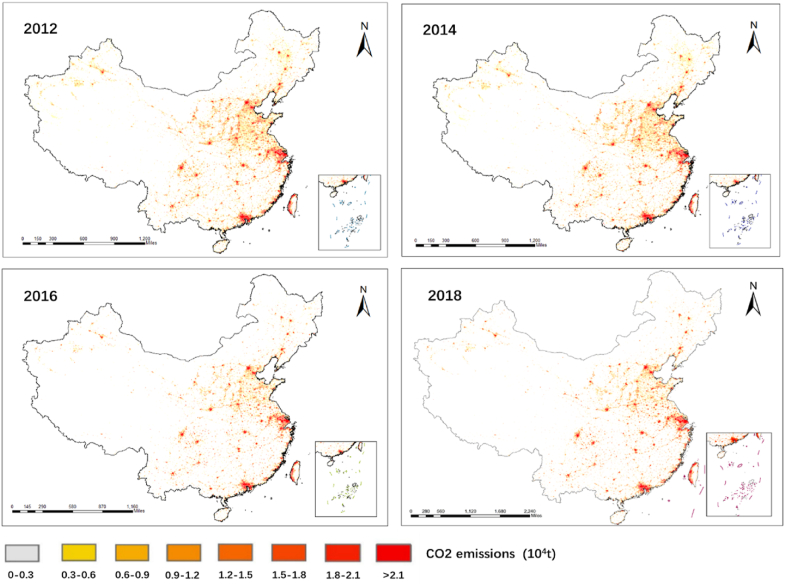
Spatiotemporal dynamics of the national CO_2_ emissions in China from 2012 to 2018.

The change of CO_2_ emissions in China from 2012 to 2019 is relatively stable, with an average annual growth rate of 2.33 % due to a number of emission reduction policies and measures promulgated by Chinese government. In 2013, the growth rate is 6.42 %. China's total CO_2_ emissions in 2015 decreased compared with that in 2014, which was the first decline in China's total CO_2_ emissions since 2001. In 2015, the growth rate of CO_2_ emissions slowed down to close to zero, and a slow increase was observed in 2017, with an average annual growth rate of 1.62 % from 2015 to 2017. In 2018 and 2019, a significant rebound in total CO_2_ emissions occurred. Fig. 5 shows the spatial and temporal distribution of the total CO_2_ emissions in China from 2012 to 2018.

Fig. 5 shows the spatiotemporal distribution of the average total CO_2_ emissions in China from 2012 to 2019. The areas with high CO_2_ emissions are mainly concentrated in the economically developed eastern coastal areas and central regions. In the eastern coastal central inland and western regions, the eastern region has a relatively high total CO_2_ emissions and the highest per capita emission intensity due to the high level of development in the eastern region and the large total energy consumption. Shanghai, Chongqing, and Tianjin have the largest emissions, accounting for 1.41 %, 1.21 %, and 1.18 % of the national average annual emissions, respectively.

**Fig. 6:**
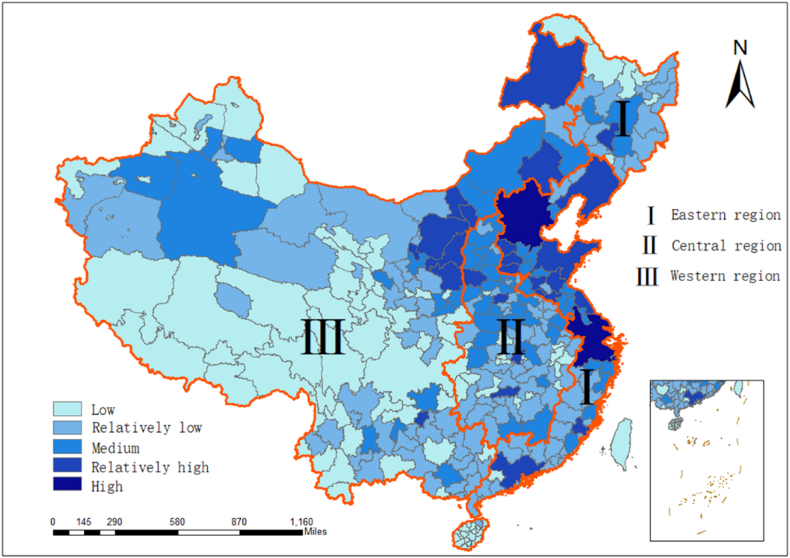
Spatiotemporal distributions of the national CO_2_ mean emissions in China from 2012 to 2019.

**Fig. 7:**
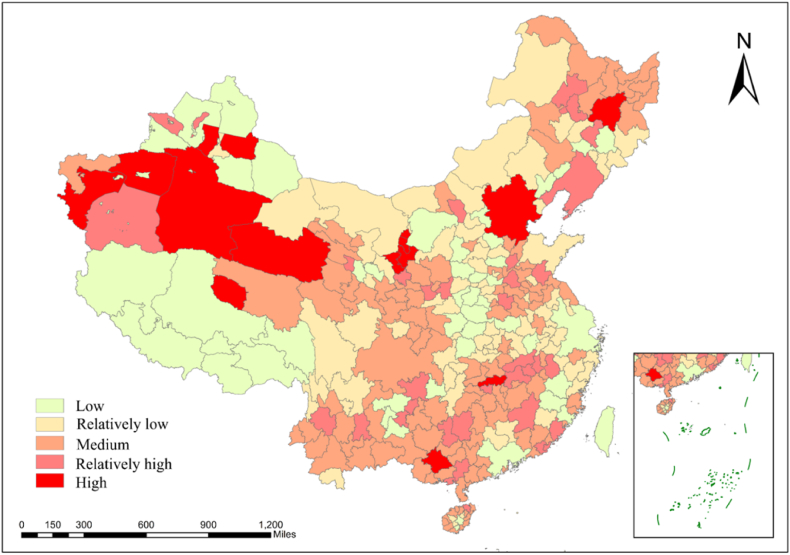
Classification of the CO_2_ emission increment speeds in China from 2012 to 2019.

Fig. 6 shows the classification of CO_2_ emission increment growth in China from 2012 to 2019. This work uses the trend value method to make a statistical calculation of the growth rate of all cities in China. The increment growth of the total CO_2_ emissions can be divided into five categories: Low growth, relatively low growth, medium growth, relatively high growth, and high growth. Fig. 7 showed that from 2012 to 2019, relatively high growth areas were mainly concentrated in the economically developed part of the central region and the western region, medium growth areas were concentrated in the central inland region, and slow growth and slow growth were mainly concentrated in the less developed areas of the central and western regions. This situation is mainly attributed to China's support for the economic development of the western region and the implementation of energy conservation and emission reduction policies in the central and eastern regions. Meanwhile, 15 cities across the country have high growth, among which Nanning in Guangxi Province, Tianjin, Changsha in Hunan Province, and some western regions are prominent.

#### 4.2.2 CO_2_ emissions spatiotemporal dynamics at the provincial scale

**Fig. 8:**
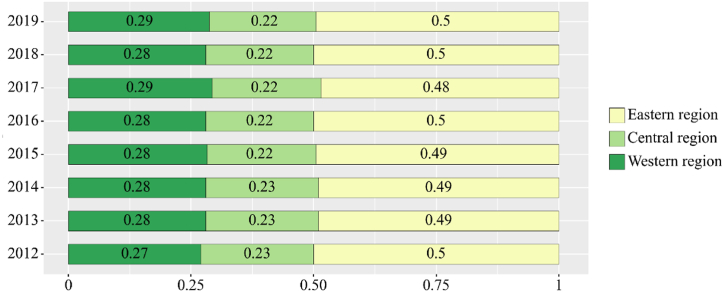
CO_2_ emission ratio of the three major economic regions to the whole country.

Fig. 8 is the CO_2_ emission ratio of the three major economic regions to the whole country. The total CO_2_ emission in central China is the highest. In 2012, the CO_2_ emission in eastern China accounted for about 42 % of the total national emissions. Shandong, Jiangsu, and Liaoning Provinces accounted for the highest proportion, which is mainly because of the high development level and high energy consumption of eastern coastal provinces. The share of the eastern region of CO_2_ emissions dropped to 38 % percent of the country in 2017 due to the implementation of energy conservation and emission reduction policies. By contrast, the proportion of CO_2_ emissions in the central region is relatively stable, accounting for 41 % of the total national emissions. In addition, the proportion of CO_2_ emissions in the western region rose from 18.9 % in 2012 to 19.2 % in 2017 due to China's economic development support for the western region. In 2018 and 2019, the CO_2_ emissions of the eastern region remained stable at around 39 %, rising compared with the previous 2 years.

**Fig. 9:**
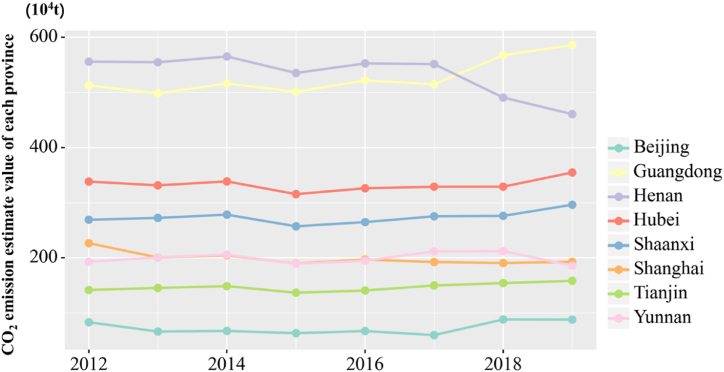
CO_2_ emissions of some representative provinces and cities from 2012 to 2019.

This work selects representative provinces and cities to analyze the trends of CO_2_ emissions in various regions during these years to further illustrate the changes in CO_2_ emissions in the three major economic zones from 2012 to 2019. The representative provinces and cities in the eastern region are Beijing, Tianjin, Shanghai, and Guangdong Province. The central region encompasses Henan and Hubei Provinces, and the western region includes Yunnan and Shaanxi Provinces. The CO_2_ emission change of each province and city during the period is shown in Fig. 9. The total CO_2_ emissions of Beijing showed a downward trend from 2012 to 2019, and that of Shanghai also depicted a downward trend. Tianjin's CO_2_ emissions significantly decreased from 2012 to 2016. The decrease in CO_2_ emissions reflects the significant effect of energy conservation and emission reduction in Tianjin, but the subsequent decrease is insignificant and stable. The main factor for this phenomenon is the economic development of Tianjin. Meanwhile, the CO_2_ emissions in Guangdong Province showed a relatively stable trend in 2012–2016, with a slight increase, and a significant increase after 2016. During the period from 2012 to 2019, the CO_2_ emissions in Henan Province significantly fluctuated, but overall, they showed a downward trend, with a significant decline. In 2013, the CO_2_ emissions in Hubei Province significantly decreased. In comparison with 2013, no significant fluctuation was observed in the following years, and an upward trend occurred in 2019. The CO_2_ emissions in Yunnan Province remained stable from 2012 to 2018, with no significant changes. A significant decline was observed in 2019. During this period, the CO_2_ emissions in Shaanxi Province rapidly increased at an average annual rate of 11.48 %. After reaching the highest value in 2017, the CO_2_ emissions of the province decreased in 2018 and rebounded in 2019. Guangdong Province showed an increase in CO_2_ emissions in 2018 and 2019. Meanwhile, Hubei and Hunan exhibited similarities, with little change in 2018 compared with 2017, but an increase in 2019. These above-mentioned data indicate that the change of CO_2_ emissions in the central region is not obvious, the CO_2_ emissions in the eastern developed region has decreased, and the CO_2_ emissions in the western region has increased.

The differential growth of CO_2_ emissions in various regions has formed a new spatial distribution pattern of CO_2_ emissions across the country. Moran's I index of national CO_2_ emissions in 2013, 2015, and 2017 are 0.3026, 0.3121, and 0.3217, respectively, based on Moran's I spatial correlation analysis of national CO_2_ emissions. These results indicate a positive correlation between the total amount of national CO_2_ emissions from 2012 to 2019, the correlation has an increasing trend, and the spatial aggregation phenomenon is becoming gradually obvious.

#### 4.2.3 CO_2_ emission spatiotemporal dynamics at the urban agglomeration scale

**Fig. 10:**
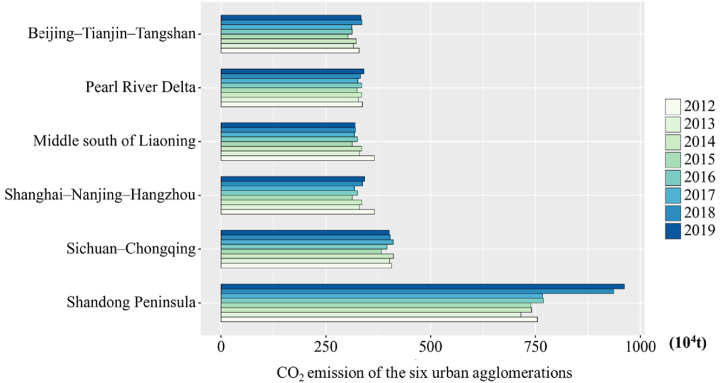
CO_2_ emission conditions of the six urban agglomerations.

Several urban agglomerations with strong comprehensive economic strength could show the comprehensive competitiveness of a country. In China, the regional economy of China has been transformed from a provincial economy to the agglomeration city economy due to factors, such as industrial upgrading, technological innovation, and factor flow. Moreover, urban agglomeration have become the main spatial form of regional development. The development of urban agglomerations could accelerate the construction of independent innovation demonstration regions and strengthen the leading role of surrounding regions. This work analyzes the CO_2_ emission spatiotemporal characteristics in six urban agglomerations, helping to more intuitively show the spatiotemporal characteristics of development of these six urban agglomerations during 2012–2019. The trend of CO_2_ emissions in the six urban agglomerations during this period is shown in Fig. 10.

The Pearl River Delta, Middle south of Liaoning, Shanghai–Nanjing–Hangzhou, and Beijing–Tianjin–Tangshan all showed negative growth. In particular, the Pearl River Delta has always been characterized by high per capita CO_2_ emissions from the beginning, but with the national economic development. These cities have vigorously developed new energy sources, significantly reduced the per capita CO_2_ emissions and showed a negative growth. Furthermore, the Beijing–Tianjin–Tangshan, Shanghai–Nanjing–Hangzhou, middle of Liaoning, and some other urban agglomerations have negative growth due to the effective measures of Chinese energy-saving and emission-reduction strategies. This phenomenon proves the effectiveness of China's energy conservation and emission reduction policies. Shandong exhibits a distinctive pattern, showing a rapid rise in 2018 and a decline in the rate of rise in 2019. The data also show that the fast-growing urban agglomerations have explored a greener and healthy development path under the premise of non-stop economic development.

## 5 Conclusion

In this work, the Flint nighttime light dataset and energy consumption dynamics are used to estimate the CO_2_ emission. The linear fitting model of these two datasets is constructed through the processing of nighttime light data and calculating of the energy statistics. The linear fitting model for these two datasets is established by processing nighttime light data and calculating energy statistics. This method could effectively compensate the shortcomings of traditional statistical method and is a relatively reliable CO_2_ emission detection method. The result of the analysis of China's CO_2_ emissions indicates that the growth rate of China's overall CO_2_ emissions is stable. In 2015, the total national CO_2_ emissions decreased as a result of the effective implementation of energy conservation and emission reduction policies. The eastern region is economically developed and has a large demand for energy. However, the total emissions in the eastern region did not significantly increase from 2012 to 2019, and that in central China did not greatly change during this period. Meanwhile, the total CO_2_ emissions in western China increased due to the state's economic support to the western region. The CO_2_ emissions variations among the three economic regions are mainly attributed to the differences in industrial structure, energy efficiency, energy structure, and industrial structure.

The results of the spatiotemporal changes of the national CO_2_ emission and nighttime light indicate that great damage has also been inflicted on the environment of China. This work proposes the following suggestions. First, the country should strengthen the supervision and administration of energy conservation and emission reduction and the management and supervision of flue gas desulfurization facilities of power plants, urban sewage treatment plants, and garbage treatment. Second, the country should improve the innovation capacity of energy conservation and emission reduction technology. Moreover, technological bottlenecks must be addressed to promote the development and the industrialization of high-efficiency energy-saving and emission reduction products in the key areas of high-tech industry. Third, the country should perform policies to form a strong incentive and constraint mechanism and establish price policies conducive to energy conservation and emission reduction. Fourth, the country should strengthen publicity to raise the national awareness of energy conservation and environmental protection. In addition, energy conservation and emission reduction publicity must be incorporated into major themed publicity activities to enhance the enthusiasm and initiative of the whole people to participate in energy conservation and emission reduction.

## CRediT authorship contribution statement

**Binbin Zhang:** Writing – review & editing, Writing – original draft. **Zongzheng Liang:** Resources. **Wenru Guo:** Visualization. **Zhanyou Cui:** Investigation, Data curation. **Deguang Li:** Writing – review & editing.

## Data availability

The data used to support the findings of this study are available from the corresponding author upon request.

## Ethics statement

Review and/or approval by an ethics committee as well as informed consent was not required for this study because this article did not involve any direct experimentation/studies on living beings.

## Declaration of competing interest

The authors declare that there is no conflict of interests regarding the publication of this article.
